# VEGF, EGFR and PSMA as possible imaging targets of lymph node metastases of urothelial carcinoma of the bladder

**DOI:** 10.1186/s12894-022-01157-7

**Published:** 2022-12-29

**Authors:** Christa Anne Maria van der Fels, Annemarie Leliveld, Henk Buikema, Marius Christianus van den Heuvel, Igle Jan de Jong

**Affiliations:** 1 Department of Urology, University Medical Center Groningen, University of Groningen, 9700 RB Groningen, The Netherlands; 2 Department of Pathology, University Medical Center Groningen, University of Groningen, 9700 RB Groningen, The Netherlands

**Keywords:** Bladder cancer, Urothelial carcinoma, Lymph nodes, Targeted imaging, Antigens

## Abstract

**Background:**

In this study we investigated the expression of vascular endothelial growth factor (VEGF), epidermal growth factor receptor (EGFR) and prostate-specific membrane antigen (PSMA) to analyze their potency as targets for the detection of lymph node (LN) metastases of urothelial carcinoma of the bladder.

**Methods:**

Antigen expression was determined in 40 samples with urothelial carcinoma and compared to 17 matched samples without metastases by immunohistochemistry. The total immunostaining score (TIS 0–12) was determined as the product of a proportion score (PS 0–4) and intensity score (IS 0–3).

**Results:**

VEGF expression was high in primary tumor and LN metastases (median TIS 8 in both) and VEGF expression was also seen in LNs without metastases (median TIS 6). EGFR expression was low in primary tumor and LN metastases (median TIS 3 and 2 respectively) and absent in LNs without metastases. PSMA expression was low in samples with urothelial carcinoma (median TIS 2).

**Conclusion:**

VEGF shows moderate to high expression levels in both primary tumors and LN metastases and could be a candidate as a target agent for imaging modalities of urothelial carcinoma. EGFR and PSMA do show low staining levels in tumor tissue with urothelial carcinoma and do not seem suitable as target agents.

*Trial registration*: The Medical Ethics Review Board of the University Medical Center Groningen approved this study on 14 December 2017 (METc UMCG 2017/639). Trial registration number (UMCG Research Register): 201700868.

## Background

Bladder cancer (BC) is the tenth most commonly diagnosed cancer for both genders worldwide and for men even the seventh [1]. Most patients present with a tumor confined to the mucosa or submucosa [2]. However, part of the tumors do show progression and become muscle invasive. Muscle invasive bladder cancer (MIBC) requires radical treatment, for example cystectomy. Different studies confirm better oncological outcome for patients in whom cystectomy is combined with lymphadenectomy versus no lymphadenectomy [3]. Moreover, survival rates improve when the total of the removed lymph nodes (LNs) increases, including the number of positive LNs [4, 5]. Adequate detection of LN involvement pre- and intraoperative could influence treatment strategy and survival rates, and is therefore urgently needed.

Computed Tomography (CT) is being used to asses LN status preoperatively. However, sensitivity is low. A recent review of Crozier et al. shows a higher sensitivity of MRI (0.60; 95% CI 0.44–0.74) and PET/CT (0.56; 95% CI 0.49–0.63), but with a wide variability across different studies [6].

The field of targeted imaging modalities for pre- and intraoperative use has rapidly evolved and has been developed in several tumor types. In a previous study we have investigated epithelial cell adhesion molecule (EpCAM) as a protein for targeted imaging in LN metastases of MIBC [7], however, molecular imaging modalities using this protein are still under development. Clinical trials were performed with imaging modalities using targets such as vascular endothelial growth factor (VEGF), epidermal growth factor receptor (EGFR) and prostate-specific membrane antigen (PSMA) in different tumor types. Various research groups investigated PSMA as a target for urothelial carcinoma with contradictory results [8, 9]. Little evidence is known about VEGF and EGFR as imaging modality targets for urothelial carcinoma.

In this study we investigated VEGF, EGFR as well as PSMA as targets for the detection of LN metastases with urothelial carcinoma of the bladder in the diagnostic setting by immunohistochemistry. Expression results on LN metastases were compared to expression of these antigens in the primary tumors. Aim of this study is to identify a candidate protein as imaging target for LN metastatic disease of urothelial carcinoma of the bladder.

## Methods

A total of 17 patients treated for urothelial cancer of the bladder were selected for this study. LN metastases (n = 23) and tumor-negative LNs (n = 17) were available in these 17 patients who all underwent LN dissection. Primary tumor samples were taken from cystectomy specimen (n = 11) during the same procedure. However, some patients appeared to have irresectable tumors and only underwent (limited) LN dissection during staging laparotomy. Primary tumor samples of these patients were used from the previously performed transurethral resection of the bladder tumor (n = 6). 16 patients were diagnosed with muscle invasive high grade urothelial carcinoma of the bladder (≥ pT2G3) and one patient had BCG refractory high grade urothelial carcinoma (pT1G3). Pathology reports showed pure UCC in 15 patients, one patient was diagnosed with a nested variant UCC and one patient had squamous differentiation in 5–10% of the cystectomy specimen besides UCC. However, only pure UCC sections were used for analysis of antigen expression. All tissue specimens were anonymously coded. The Medical Ethics Review Board of the University Medical Center Groningen approved this study on 14 December 2017 (METc UMCG 2017/639). Trial registration number (UMCG Research Register): 201700868.

VEGF and PSMA expression on the primary tumor, LN metastases and tumor-negative LNs were determined by immunohistochemistry on 4 µm-thick paraffin embedded slides. Slides were deparaffinized with xylene baths and decreasing grades of alcohol. Antigen retrieval was performed by heating microwave (500 W) for 15 min in a 10 mM citrate buffer at pH 6.0, with a cooldown period for 15 min afterwards. Endogeneous peroxidase was blocked with 0.3% hydrogen peroxide in PBS for 20 min in the dark. Slides were incubated with the primary antibodies, diluted in 1% BSA/PBS for 1 h at room temperature with mouse monoclonal antibody (AB) anti-PSMA (1:50, YPSMA-1, clone sc-59674, Santa Cruz Biotechnology, Santa Cruz, CA, USA), and mouse monoclonal AB anti-VEGF (C-1) (1:100, clone sc-7269, Santa Cruz Biotechnology, Santa Cruz, CA, USA). In the secondary step, slides were incubated with rabbit anti-mouse AB conjugated to polymer-horseradish peroxidase (DAKO, Glostrup, Denmark), diluted at 1:100 in 1% BSA/PBS with 1% AB serum. In the tertiary step goat anti-rabbit AB conjugated to polymer-horseradish peroxidase (DAKO, Glostrup, Denmark) was used, diluted at 1:100 in 1% BSA/PBS with 1% AB serum. Secondary and tertiary antibodies were incubated for 30 min at room temperature. After every step, slides were washed with PBS and dried. Next, slides were immersed for 10 min in a solution of 0.05% 3,3′-diaminobenzidine (Sigma-Aldrich, Steinheim, Germany) and 0.03% hydrogen peroxide in PBS in the dark for visualization of the signal as brown staining. After washing with demineralized water, slides were slightly counterstained with haematoxylin, dehydrated by increased grades of alcohol and when dried, mounted with Tissue Tek film (Sakura Finetek, Leiden, The Netherlands).

Staining of the antigens was compared to the staining of Pan-Cytokeratin (CK AE1/AE3), an epithelial marker that is positive in the vast majority of UCC of the bladder, to secure the accuracy and reliability of our project.

For CK AE1/AE3 and EGFR, immunohistochemistry was performed in the Autostainer Link 48 (Dako). This includes antigen retrieval (4 min) in protease 3, incubation with the primary antibody (16 min for anti-CK AE1/AE3; 8 min for anti-EGFR) and incubation with the visualization complex (8 min). Counterstaining was performed with haematoxylin.

The immunostaining was evaluated in a previously described manner [10], by multiplying the proportion score (PS) by the intensity score (IS), resulting in the total immunostaining (TIS). The PS, representing the proportion of the tumor positive for an antigen staining, was scored as 0, none; 1, < 10%; 2, 10–50%; 3, 51–80%; 4, > 80%. This was eye-ball evaluated by an experienced genitourinary pathologist (M.H.). The IS, representing the intensity of immunostaining, was scored as 0, no staining; 1, weak; 2, moderate; 3, strong. As IS scoring may be subject to intraobserver variability, IS was scored digitally using the Visiopharm Intergrator System (VIS) (version 2020.02.0.7219, Hørsholm, Denmark). Slides were scanned by the Philips Ultrafast Scanner 1.6, at 40 times magnification.

Descriptive analyses were used to describe the results and are shown as median values with interquartile range. SPSS statistics (version 23.0 for Windows, IBM Corp, Armonk, NY, USA) was used for analyses.

## Results

**Fig. 1:**
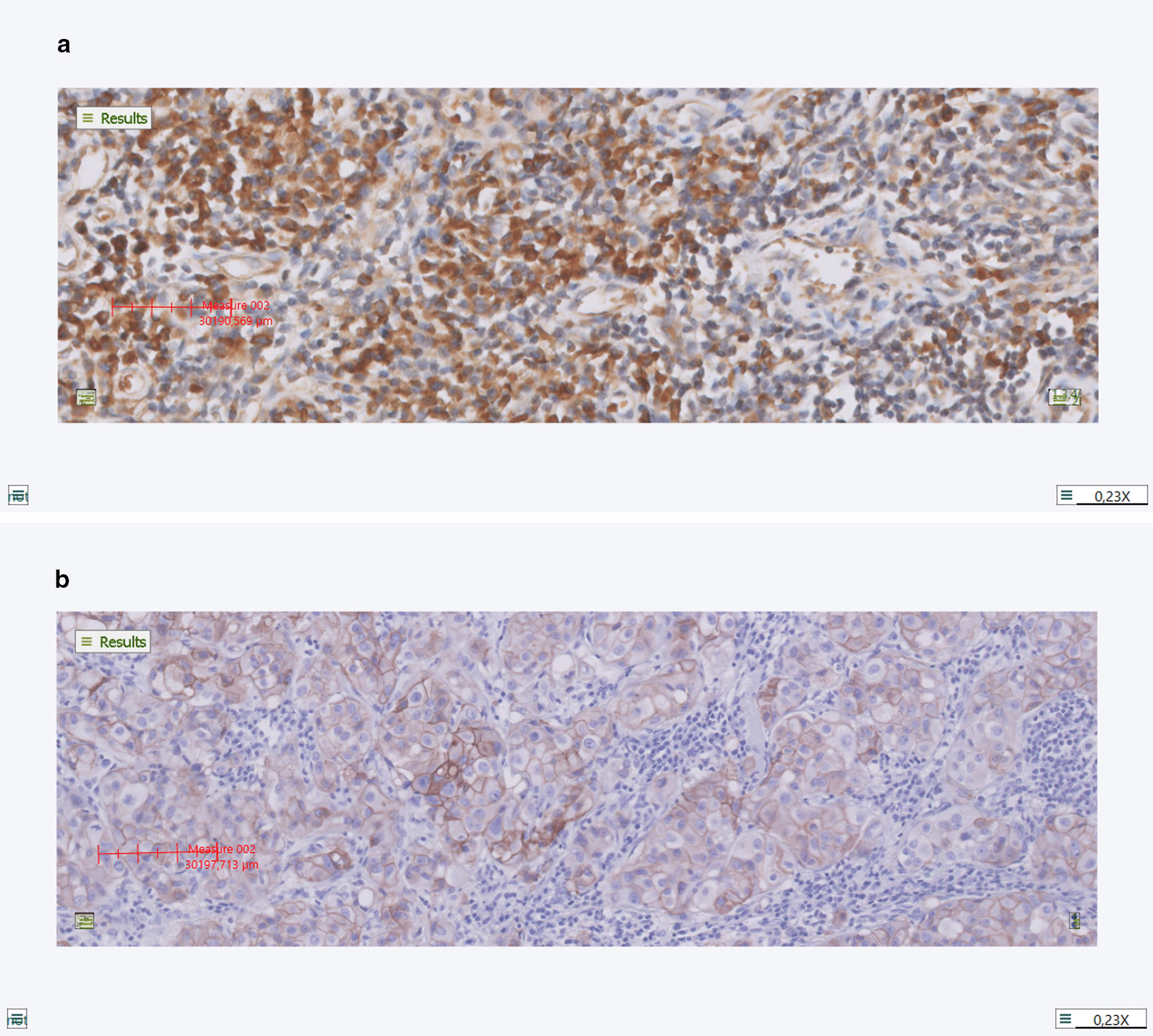
(**a**) VEGF expression, IS 2; (**b**) EGFR expression, IS 1. Intensity of the staining was scored digitally using the Visiopharm Intergrator System (VIS) (version 2020.02.0.7219, Hørsholm, Denmark). Slides were scanned by the Philips Ultrafast Scanner 1.6, at a 40 × magnification

VEGF expression was prominent in LN metastases with urothelial carcinoma and also in primary tumors. A maximum proportion score (PS) of VEGF was seen in LN metastases with urothelial carcinoma and in primary tumors (median PS 4). Median intensity score (IS) in both LN metastases and primary tumors was 2. Figure 1a. This resulted in a median total immunostaining score (TIS) of 8 in all tumor positive samples (Table 1). VEGF expression was absent in 3/17 (18%) primary tumors and 1/22 (5%) LN metastases. Background staining of VEGF was presented in all negative LNs. Median TIS in lymphoid tissue of LNs without metastases was 6 (Table 1). This resulted in a ratio between tumor-to-no tumor tissue of 1.3 ± 0.7 (median ± SD).

**Table 1:** Scoring immunoreactivity of all antibodies on urothelial carcinoma, median (interquartile range)

	VEGF		EGFR		PSMA		CK AE1/AE3	
	*n*	TIS (IQR)	*n*	TIS (IQR)	*n*	TIS (IQR)	*n*	TIS (IQR)
Primary tumor	17	8 (7–8)	17	3 (2–4)	12	2 (0.25–2)	17	12 (12–12)
LN metastases	22	8 (4–9)	23	2 (0–4)	13	2 (0.5–2)	23	12 (12–12)
Negative LNs	17	6 (3.5–6)	17	0 (0–0)				

TIS, total immunostaining score; IQR, interquartile range

The proportion score of EGFR in urothelial carcinoma was high in most of the samples. However, the intensity score was low (median IS 1 in both LN metastases and primary tumors). Figure 1b. This resulted in low EGFR expression in LN metastases (median TIS 2) and in primary tumors (median TIS 3). EGFR expression was absent in only 2/17 (12%) primary tumors and in 5/23 (22%) LN metastases with urothelial carcinoma. LNs without tumor did not show EGFR expression (median TIS 0). Table 1.

Because of the contradictory results of PSMA as target for BC in other studies, a pilot of 12 patients was used (Table 1). Very low or absent expression of PSMA was seen in both LN metastases and in the primary tumors. Therefore, no further samples were tested for PSMA expression.

CK AE1/AE3 was used as a control for epithelial origin of the tumors. Maximum CK AE1/AE3 expression was seen in primary tumors and metastatic LNs (Table 1). No expression of CK AE1/AE3 was seen in tissue without tumor in these samples.

## Discussion

Previously we showed that EpCAM has a high tumor distinctiveness for LN metastases with urothelial carcinoma [7]. In the current study we present our results of the expression of the more commonly used biomarkers VEGF, EGFR and PSMA on LN metastases with urothelial carcinoma. VEGF expression was presented in LN metastases and in primary urothelial carcinoma of the bladder. In addition, lymphoid tissue without tumor showed VEGF expression, although in a lower intensity. EGFR and PSMA do not seem suitable as imaging targets for urothelial carcinoma of the bladder because of the low staining results of these antigens in the current study.

We compared our results to the available literature of expression of VEGF, EGFR and PSMA on urothelial carcinoma of the bladder. 82% of the primary tumors and 95% of the LN metastases investigated in this study showed moderate to high VEGF expression. Expression of VEGF was less prominent presented in negative LNs. Zaravinos et al. reported higher transcript levels of VEGF in bladder cancer tissue than in normal urothelium, and VEGF expression was higher in grade I/II versus grade III tumors [11]. Other research groups reported VEGF expression in 58–86% of urothelial carcinoma specimens. In these studies, VEGF was not correlated to prognosis of patients with urothelial carcinoma of the bladder and VEGF expression was not evaluated in normal urothelium [12, 13].

In our study, 88% of the primary tumors and 78% of the LN metastases showed EGFR expression, however, most of our samples showed only weak to moderate expression. In comparison, Chiang et al. reported moderate to strong EGFR expression in 37% of the 39 patients with MIBC [14]. Another research group reported EGFR expression in 55.4% of all patients with urothelial bladder cancer. The expression level of EGFR was significant higher in samples with higher stage and grade urothelial carcinoma versus lower stage and grade. However, most of the samples showed only weak to moderate EGFR expression, as in our study [15]. On the other hand, Carlsson et al. reported EGFR expression in 71% of primary bladder tumors and in 69% of corresponding metastases. Positive EGFR expression was reported when ≥ 10% of the tumor cells were stained with a moderate to strong intensity score [16]. An explanation for the strong staining intensity in the samples of this study was not described. Only high grade tumors were evaluated in the study of Carlsson et al., comparable with the urothelial carcinomas in our study.

Gala et al. showed very low staining intensity of PSMA in normal and malignant urothelial specimens in contrast to high staining intensity of PSMA in prostate cancer cell lines [17]. On the other hand, immunohistochemical analysis of Schreiber et al. demonstrated PSMA expression in a high number of patients with urothelial carcinoma (78.7%) [8]. However, only the fraction of stained tissue was graded in this study, not the degree or intensity of the staining.

Urothelial carcinoma of the bladder is a heterogeneous disease and different biomarkers are expressed in low grade urothelial carcinoma vs high grade urothelial carcinoma or metastatic disease [18]. Only limited number of biomarkers could be used as targets for the detection of tumor tissue [19]. In this respect, finding a biomarker suitable as imaging target for LN metastases of urothelial carcinoma of the bladder is challenging. VEGF is an endothelial marker that is widely used as imaging agent. VEGF is associated with angiogenesis and endothelial cell growth [11] and is expressed in normal lymphoid tissue as well. Heterogeneity of VEGF uptake using SPECT- and PET-imaging modalities in different tumors has been reported before. In these studies, VEGF uptake was seen in tumor tissue as well as in normal tissue and well perfused organs like heart and liver. However, in individual patients, VEGF uptake was always higher in tumor tissue than in normal tissue, leading to high tumor detection rates. Moreover, VEGF uptake in the circulation decreased over time, whereas VEGF uptake in tumor increased. Increasing tumor to background ratios were seen over time using these imaging modalities. VEGF uptake in imaging modalities was positively correlated with the VEGF expression measured by IHC in tumor tissue versus normal in these studies [20, 21]. VEGF might be potentially valuable for a proper detection of LN metastatic bladder cancer in the diagnostic setting. Pre-operatively diagnosed LN metastatic disease could change disease management in non-surgically options. On the other hand, intraoperative detection of limited positive LNs could lead to a better removal of these positive LNs and improve survival rates [4, 5].

Since EpCAM and EGFR act on epithelial cells, no expression of these proteins is found in LNs without tumor. However, surrounding normal tissue as endometrium and prostate do show EGFR reactivity, thought predominant localization of the protein is seen in the cytoplasm [22]. Since effective targeting by imaging modalities is mainly based on membrane-bound extracellular targets [18], this might not be a problem. Similar to VEGF, the reported expression of EGFR in urothelial carcinoma of the bladder has a wide range [14–16]. A heterogeneous EGFR expression is also seen in other tumors as head and neck cancer tissues. However, real-time imaging studies using EGFR as target report consistent tumor-to-background ranges in these tissues [23, 24].

In our study, we used a small sample size. However, in contrast to other studies, we investigated a homogeneous patient population. All patients had high grade, metastasized, urothelial carcinomas of the bladder and we did not see a wide variation in staining results between these patients. Therefore, our results might be representative for other patients with MIBC.

## Conclusions

VEGF shows moderate to high expression levels in both primary tumors and LN metastases. Therefore VEGF could be a potential candidate as a target agent for imaging modalities of urothelial carcinoma of the bladder in order to achieve a more proper disease staging. Due to low intensity staining levels in both primary urothelial carcinoma and metastases, EGFR and PSMA are not suitable as target agents for imaging modalities.

## Data Availability

The datasets used and/or analysed during the current study are available from the corresponding author on reasonable request.

## Abbreviations

AB: Antibody
BC: Bladder cancer
CT: Computed tomography
EGFR: Epidermal growth factor receptor
EpCAM: Epithelial cell adhesion molecule
IS: Intensity score
LN: Lymph node
MIBC: Muscle invasive bladder cancer
PSMA: Prostate-specific membrane antigen
PS: Proportion score
TIS: Total immunostaining score
UCC: Urothelial cell carcinoma
VEGF: Vascular endothelial growth factor
